# Supplementary material for: SyNDI: synchronous network data integration framework
Source: BMC Bioinformatics. 2018 Nov 6;19:403. doi: 10.1186/s12859-018-2426-5 (PMC6219086; doi:10.1186/s12859-018-2426-5)
Supplement: Supplementary file 5 — Files of the example presented in Additional file 4. This additional file is a zip package that contains all files used in Additional file 4. Detailed descriptions of each of these files are presented in a README file included in this zip package. (GZ 30411 kb) [file 12859_2018_2426_MOESM5_ESM.gz › SyNDITools/Meme2Fimo/meme_4.9.0_4.tar.gz!/meme_4.9.0/website/html/mhmm-download.in]

Meta-MEME v3.3


> ### Download
>
> ---
>
> The Meta-MEME software is available for interactive use via the web.
> Alternatively, you can download the software for installation
> elsewhere. Each software distribution comes with the complete
> source code as well as program binaries for a particular hardware
> architecture:
>
>
>
> Release Notes.
>
> Installation Instructions.
>
> Not sure which platform you have? Try typing 'uname -m' on your
> system and matching the resulting output to one of the
> parenthesized words in the links above.
>
> If you don't see your platform listed, you may download the source
> only distribution, but you will need to compile the program on your
> system. I would appreciate receiving copies of the program binaries
> for any platform not listed above, so that I can create a software
> distribution for that platform.
>
>
>
> ---
>
> Return to the Meta-MEME home page.
>
> *Please send comments and questions to:
> @METAMEME\_CONTACT@.*
